# Induction of Triploid Grass Carp (*Ctenopharyngodon idella*) and Changes in Embryonic Transcriptome

**DOI:** 10.3390/ani15152165

**Published:** 2025-07-22

**Authors:** Zixuan E, Han Wen, Yingshi Tang, Mingqing Zhang, Yaorong Wang, Shujia Liao, Kejun Chen, Danqi Lu, Haoran Lin, Wen Huang, Xiaoying Chen, Yong Zhang, Shuisheng Li

**Affiliations:** 1 State Key Laboratory of Biocontrol and School of Life Sciences, Southern Marine Science and Engineering Guangdong Laboratory (Zhuhai), Guangdong Provincial Key Laboratory for Aquatic Economic Animals, Guangdong Provincial Engineering Technology Research Center for Healthy Breeding of Important Economic Fish, Institute of Aquatic Economic Animals, Sun Yat-Sen University, Guangzhou 510275, China; ezix@mail2.sysu.edu.cn (Z.E.); wenh37@mail2.sysu.edu (H.W.); tangysh6@mail2.sysu.edu.cn (Y.T.); zhangmq58@mail2.sysu.edu.cn (M.Z.); wangyr95@mail2.sysu.edu.cn (Y.W.); liaoshj6@mail2.sysu.edu.cn (S.L.); chenkj36@mail2.sysu.edu.cn (K.C.); ludanqi@mail.sysu.edu.cn (D.L.); lsslhr@mail.sysu.edu.cn (H.L.); 2 Key Laboratory of Animal Nutrition and Feed Science in South China, Guangdong Provincial Key Laboratory of Animal Breeding and Nutrition, Institute of Animal Science, Ministry of Agriculture and Rural Affairs, Guangdong Academy of Agricultural Sciences, Guangzhou 510640, China; huangwen549@126.com (W.H.); xiaoyingchen05@126.com (X.C.); 3 Laboratory for Marine Fisheries Science and Food Production Processes, Qingdao Marine Science and Technology Center, Qingdao 266237, China

**Keywords:** grass carp, triploidy, cold shock, embryonic transcriptome

## Abstract

Grass carp is an economically important cultured species in China. Triploid embryo production is widely applied in aquaculture to achieve reproductive sterility, improve somatic growth, and reduce ecological risks associated with uncontrolled breeding. In this study, a simple cold shock method for inducing triploid grass carp was developed. The triploid induction rate of 71.73 ± 5.00% was achieved by applying a cold treatment at 4 °C for 12 min, starting 2 min after artificial fertilization. Flow cytometry and karyotype analysis revealed that triploid individuals exhibited a 1.5-fold increase in DNA content compared to diploid counterparts, with a chromosomal composition of 3n = 72 (33m + 36sm + 3st). Additionally, embryonic transcriptome analysis demonstrated that, in the cold shock-induced embryos, genes associated with abnormal mesoderm and dorsal–ventral axis formation, zygotic genome activation (ZGA), and anti-apoptosis were downregulated, whereas pro-apoptotic genes were upregulated, which may contribute to the higher abnormal mortality observed during embryonic development. Overall, this study demonstrates optimized conditions for inducing triploidy in grass carp via cold shock and provides insights into the transcriptomic changes that take place in cold shock-induced embryos, which could inform future grass carp genetic breeding programs.

## Simple Summary

Triploidy induction is a widely used method for producing sterile fish populations, which offers significant advantages for both aquaculture and fishery management. Grass carp (*Ctenopharyngodon idella*), an economically important species in China, is extensively cultured in major river systems of Eastern Asia. In this study, a straightforward method for inducing triploidy in grass carp using cold shock was developed. This approach successfully induced approximately 300,000 triploid larvae at a grass carp farm, demonstrating its practical applicability in aquaculture settings. Furthermore, we conducted an embryonic transcriptome analysis, revealing the downregulation of genes involved in mesoderm, and dorsal–ventral axis formation, zygotic genome activation (ZGA), and anti-apoptosis were downregulated, whereas pro-apoptotic genes were upregulated in cold shock-induced embryos. These molecular changes may contribute to the elevated mortality rates observed during embryonic development, providing valuable insights into the mechanisms behind inducing cold shock in embryos.

## 1. Introduction

Grass carp (*Ctenopharyngodon idella*), a common cyprinid fish, is widely distributed in the major river systems of Eastern Asia [1]. Known for its herbivorous diet, grass carp is characterized by lower protein requirements and rapid growth, making it highly favored by farmers [2]. Additionally, its nutritious and flavorful flesh has made it popular among consumers [3]. The global production of grass carp through farming has steadily increased in recent years, totaling 5791.5 kilotons in 2022 [1]. Beyond aquaculture, grass carp is also utilized as a biological control organism for aquatic weeds [4]. However, the absence of natural predators has led to grass carp overpopulation in certain regions, disrupting local aquatic ecosystems, particularly in North America [5].

Infertility control in fish breeding is a major focus of aquatic research, with sterile fish being regarded as a means to redirect reproductive energy into somatic growth, thereby enhancing fish production in aquaculture [6,7]. The introduction of sterile fish can also prevent natural spawning in new aquatic ecosystems, reducing ecological risks [8]. In addition to triploidization, other strategies such as monosex culture and hybridization have also been employed to achieve reproductive containment in aquaculture species. Triploidization in fish is recognized as an ideal strategy for generating infertile populations, offering benefits for both aquaculture practices and ecological control in fisheries [9]. Triploids can be generated using two main methods. First, physical shocks (hydrostatic pressure or thermal shocks) or chemical treatments suppress the emission of the second polar body (PBII) in diploid zygotes [10,11]. The physical shock method has been extensively used in fish species, such as *Rhamdia quelen* and *Nibea mitsukurii* with cold shock [12,13], *Lota lota* [14] and *Oreochromis mossambicus* with heat shock [14,15], and *Megalobrama amblycephala* and *Siniperca chuatsi* with hydrostatic pressure [16,17]. Second, distant hybridization is an effective approach to producing allotriploids, particularly in cyprinids [18]. Successful allotriploid hybrids have been produced through different hybridization combinations such as *M. amblycephala* ♀ × *Xenocypris davidi* Bleeker ♂ and *M. amblycephala* ♀ × *Erythroculter ilishaeformis* ♂ [18,19]. Artificial triploid induction in grass carp via temperature shock and hydrostatic pressure has also been reported [20,21], but these methods have not been widely adopted in aquaculture, particularly in China, likely due to the challenging induction conditions in commercial fish farms.

Fish are highly sensitive to physical shocks during embryonic development. Studies have shown that induced embryos exhibit high mortality rates during development [22]. Physical treatments can disrupt gene expression, hinder mitotic division and proliferation in polyploid cells, and lead to aberrant chromosome segregation and aneuploidy [23,24]. However, most studies focus on the effects of external environmental factors on embryonic development, with little attention given to the global gene expression profiles of embryos during development.

In this study, an optimized cold shock method was developed for inducing triploidy in grass carp, which is more suitable for practical application in fish farms. Compared to conventional approaches, the optimal duration of cold treatment is precisely defined in the optimized cold shock protocol developed in this study, resulting in a significantly higher triploid induction rate. The ploidy of the larvae and juveniles was identified through flow cytometry and karyotype analysis, and a transcriptome analysis of cold shock-induced embryos during development was performed using RNA sequencing technology.

## 2. Materials and Methods

### 2.1. Broodstock Cultivation and Breeding

Grass carp were reared at the Jianglong Shunjing Aquaculture Farm in Zhongshan, China. Sexually mature females and males (approximately 4 years old, total length 70–75 cm, body weight 4.2 ± 0.3 kg) were selected for their first induced spawning. These broodstock were administered a hormonal regimen to induce gamete production. The regimen consisted of an initial injection of luteinizing hormone-releasing hormone analogue (LRH-A) at a dose of 6 μg/kg, followed by a second injection of LRH-A (15 μg/kg) and domperidone (DOM; 3 mg/kg) 8–9 h later. Eggs and sperm were obtained by gently squeezing the abdomen of the broodstocks. After artificial insemination, the eggs were cold-shocked for triploid induction. The animal experiments were certified by the Committee of Animal Research and Ethics for Sun Yat-sen University.

### 2.2. Artificial Induction with Cold Shock

The embryos hatched in pond water maintained at 28 ± 1 °C, with a pH of 6.8–7.2 and stable dissolved oxygen concentrations (6.0 to 8.0 mg/L). Based on a previous study that successfully induced gynogenesis in grass carp with cold shock (4–6 °C) [25], the cold shock temperature in this study was set at 4 °C, applied 2 min after fertilization, with shock durations of 10, 12, and 14 min evaluated. The cleavage, fertilization, and hatching rates were recorded at the 2-cell, blastula, and hatching stages, respectively. The following calculation formulas were used:

Cleavage rate (%) = (the cleavage embryos/total eggs) × 100%;

Fertilization rate (%) = (the fertilization embryos/total eggs) × 100%;

Hatching rate (%) = (the live larvae/total eggs) × 100%.

### 2.3. Embryogenesis Analysis

After fertilization, the eggs of both the diploid and cold shock-induced groups were collected. Embryogenesis was monitored under a Leica EZ4W stereomicroscope (Leica, Wetzlar, Germany). The survival rates of developmental embryos were calculated, and the procedure was replicated three times independently. The calculation formula used was as follows:

Survival rate (%) = (the live embryos/total eggs) × 100%.

### 2.4. Flow Cytometry Analysis and Karyotype Analysis

Approximately 30 larvae (embryonic hatching stage) from each treatment group were selected for flow cytometry analysis. The tissue was incubated with the nuclei extraction buffer for 20 min at 25 °C and subsequently incubated in the dark with a staining solution containing RNase and propidium iodide for 30 min. The DNA content of each larva was estimated using a Cytomics FC-500 flow cytometer (Beckman Coulter, Brea, CA, USA).

Juvenile fish (3 months post-fertilization) were selected for karyotype analysis. Chromosome preparations were made using head kidney tissue according to the protocol in a published study [11]. The number and type of chromosomes were analyzed under a Nikon Eclipse Ni-E microscope (Nikon, Tokyo, Japan).

### 2.5. Sample Collection, RNA Preparation, Library Construction, and Sequencing

Embryos at the blastula and gastrula stages from the diploid and cold shock-induced groups were collected in triplicate biological sets (50 embryos per replicate). Total RNA was isolated using TRIzol reagent (Thermo Fisher Scientific, Waltham, MA, USA). After the quality assessment, mRNA was enriched using poly(A) selection magnetic beads and fragmented by heat. Using random primers, first-strand cDNA synthesis was performed via reverse transcription, and then the second cDNA strand was synthesized by employing a PCR reaction. The cDNA was isolated using a PCR Extraction Kit (Qiagen, Venlo, The Netherlands) and finally ligated to the Illumina adapters for sequencing. Target fragments were collected using Hieff NGS^®^ DNA Selection Beads (Yeasen Biotechnology Co., Ltd., Shanghai, China), followed by library amplification and sequencing on the Illumina NovaSeq X Plus (Illumina, San Diego, CA, USA).

### 2.6. Differential Gene Expression Analysis

Raw sequencing data were processed using a standardized bioinformatics pipeline. Fastp (v0.18.0) was used for adapter trimming and low-quality read filtering [26]. Bowtie2 was then employed to align reads to the SILVA rRNA database, and mapped reads were removed to eliminate ribosomal RNA [27]. The clean reads were subsequently aligned to the *Ctenopharyngodon idella* reference genome (NCBI accession: PRJNA745929) using HISAT2 (v2.1.0) [28]. Transcript assembly and expression levels were carried out with StringTie (v1.3.1) and RSEM (v1.3.3), respectively [29,30]. Principal component analysis (PCA) was conducted by applying the R package gmodels (v2.18.1) (http://www.r-project.org/; accessed on 25 June 2018).

Differential gene expression between diploid and triploid embryos was analyzed using DESeq2 (v1.38.3) for group-level comparisons and edgeR (v3.40.2) for pairwise analyses [29,31]. Differentially expressed genes (DEGs) were screened when the genes met stringent thresholds (|log2FC|) of ≥1 and FDR-adjusted *p* < 0.05). Functional enrichment analysis was performed using Gene Ontology (GO) term annotation from the GO database (http://www.geneontology.org; accessed on 10 February 2022) [32] and Kyoto Encyclopedia of Genes and Genomes (KEGG) pathway database [33]. Significantly enriched terms and pathways were identified based on an FDR threshold of < 0.05.

### 2.7. Quantitative Real-Time Polymerase Chain Reaction (qRT-PCR)

Ten genes—bone morphogenetic protein 2b (*bmp2b*), bone morphogenetic protein 4 (*bmp4*), gremlin 1b (*grem1b*), wingless-type MMTV integration site family member 5b (*wnt5b*), frizzled class receptor 8b (*fzd8b*), SMAD family member 4a (*smad4*), frizzled class receptor 1 (*fzd1*), bone morphogenetic protein receptor type-1A-like (*bmpr1a*), frizzled class receptor 9b (*fzd9b*), and wingless-type MMTV integration site family member 8a (*wnt8a*)—were selected to verify the transcriptome data. The specific primer pairs that were designed are listed in Supplementary Table S1. Using the SYBR qPCR Master Mix (Vazyme, Nanjing, China), qRT-PCR was performed using a LightCycler 480 II instrument (Roche, Basel, Switzerland) according to our previous program [34]. The gene expression was normalized against *β-actin*, and the data were calculated using the 2^−ΔΔCT^ method.

### 2.8. Statistical Analysis

Data are presented as means ± standard deviation (SD). Statistical difference analysis was conducted by unpaired Student’s *t* tests or one-way ANOVA, followed by Tukey’s test, and *p* < 0.05 was estimated statistically significant. Prior to statistical testing, data were assessed for normality using the Shapiro–Wilk test and for homogeneity of variances using Levene’s test.

## 3. Results

### 3.1. Induction Conditions for Triploid Grass Carp

Our analysis of fertilization success, hatching efficiency, and triploid induction rates under varying cold shock conditions revealed distinct outcomes (Table 1). When initiated 2 min post-fertilization (mpf), Treatment 1 (4 °C for 10 min) yielded the highest fertilization (61.52 ± 1.03%) and hatching rates (12.83 ± 0.77%) but a triploid rate of only 10 ± 7.13%. Treatment 2 (4 °C for 12 min) produced the highest triploid rate (71.73 ± 5.00%) compared to other treatments, while no triploids were detected in Treatment 3 (4 °C for 14 min). Based on the induction conditions of Treatment 2, approximately 300,000 triploid larvae were successfully induced at the grass carp farm in Zhongshan, China, in 2024, indicating that this method is viable for large-scale aquaculture applications.

### 3.2. Investigation of Embryonic Development in Cold Shock-Induced Grass Carp

**Figure 1:**
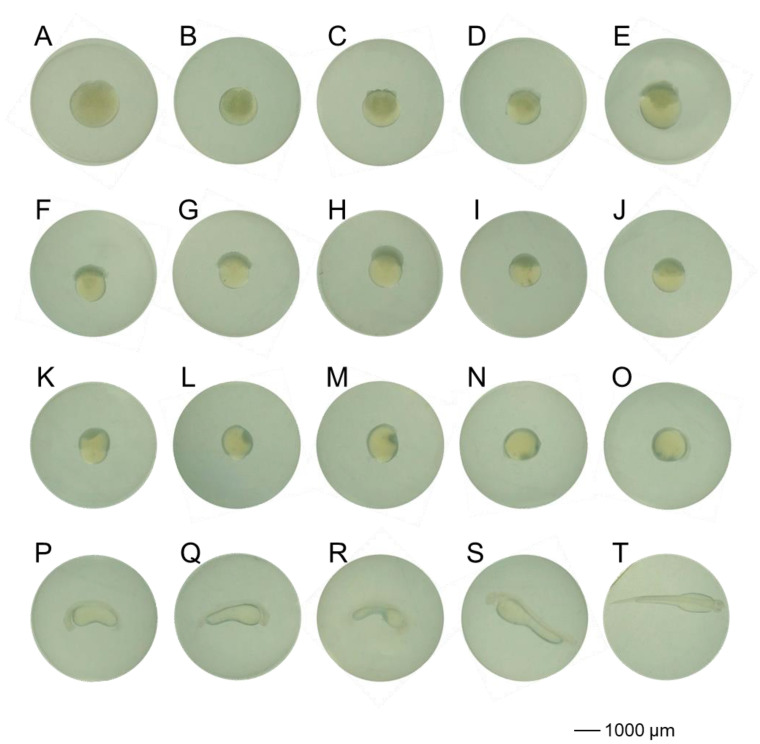
Morphology of embryonic development in cold shock-induced grass carp. (**A**): 2-cell stage; (**B**): 4-cell stage; (**C**): 8-cell stage; (**D**): 16-cell stage; (**E**): 32-cell stage; (**F**): 64-cell stage; (**G**): early blastula stage; (**H**): mid-blastula stage; (**I**): late blastula stage; (**J**): early gastrula stage; (**K**): mid-gastrula stage; (**L**): late gastrula stage; (**M**): neurulation stage; (**N**): blastopore closure stage; (**O**): somite segmentation stage; (**P**): optic vesicle formation stage; (**Q**): tail bud stage; (**R**): muscular contraction stage; (**S**): pre-hatching stage; (**T**): embryo hatching stage.

**Figure 2:**
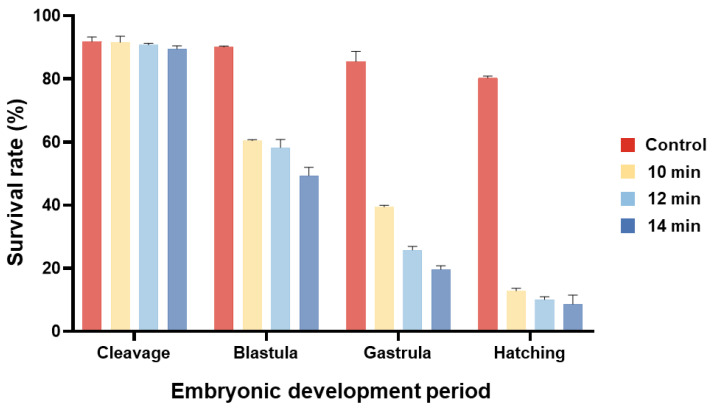
Survival rate of grass carp during embryonic development.

The embryonic development of the cold shock-induced group was characterized as follows: The first cleavage occurred at 0.7 h post-fertilization (hpf), initiating the cleavage period with systematically arranged blastomeres (Figure 1A–F). At 2.4 hpf, blastocoel formation marked the onset of the blastula stage (Figure 1G–I). Gastrulation began at 5.4 hpf, characterized by the migration of marginal cells along the yolk periphery toward the vegetal pole (Figure 1J–L). Organogenesis was completed at 10.4 hpf, followed by the initiation of rhythmic muscular contractions at 16.3 hpf (Figure 1M–P). Embryonic hatching began at 21.3 hpf (Figure 1T). The development of artificially induced embryos exhibited no significant differences in morphology or developmental timing compared to diploid embryos; however, triploids demonstrated significantly higher mortality rates during the blastula and gastrula stages compared to diploid embryos (Supplementary Table S2, Figure 2).

### 3.3. Analysis of DNA Content and Karyotype

**Figure 3:**
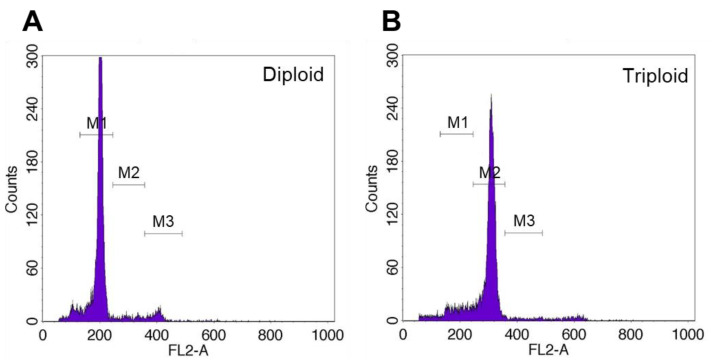
Flow cytometric analysis of diploid larvae and cold shock-induced triploid larvae. (**A**): DNA content of diploids. (**B**): DNA content of triploids.

**Figure 4:**
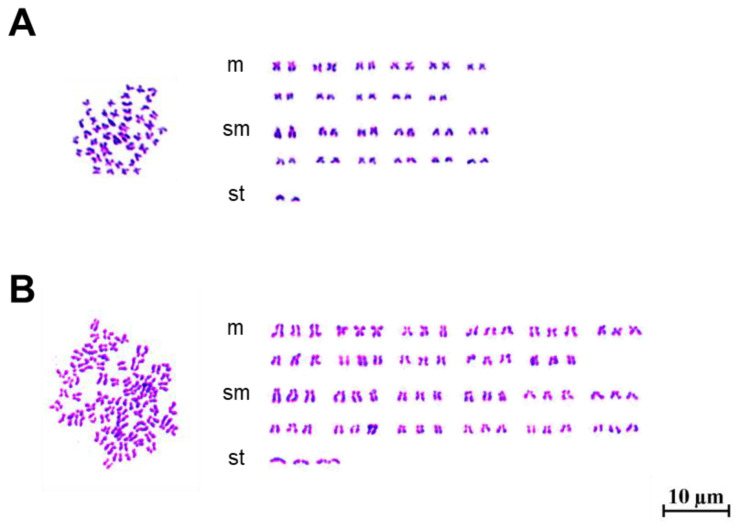
Chromosome counting and karyotype analysis of diploid and triploid individuals. (**A**): Karyotype and chromosome number of diploid individuals. (**B**): Karyotype and chromosome number of triploid individuals.

The DNA content in diploid and triploid grass carp was quantified, revealing DNA concentrations of approximately 200 for diploids and 300 for triploids (Figure 3). Triploid grass carp exhibited a 1.5-fold higher DNA content than diploids. Karyotype analysis revealed that diploid individuals (2n) possessed 48 chromosomes, corresponding to a karyotype formula of 2n = 48 =22 metacentric (m) + 24submetacentric (sm) + 2subtelocentric (st) (Figure 4A). In contrast, triploid individuals (3n) exhibited 72 chromosomes, with a karyotype formula of 3n = 72 = 33m + 36sm + 3st (Figure 4B), demonstrating an entire additional chromosomal set compared to diploids.

### 3.4. Analysis of Transcriptome Data

**Figure 5:**
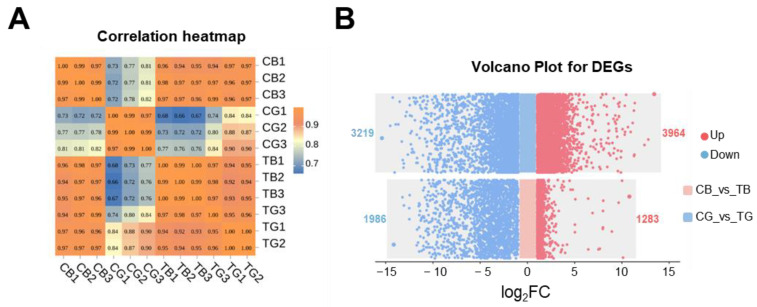
Correlation analysis of DEGs at the blastula and gastrula stages of diploid (CB and CG) and cold shock-induced (TB and TG) embryos. (**A**): Associated heatmaps of each sample. (**B**): Volcano plot derived from the DEGs of each stage.

Twelve cDNA libraries were generated using diploid and cold shock-induced embryos at the blastula and gastrula stages. Raw sequencing data were submitted to the NCBI Sequence Read Archive (accession numbers: PRJNA1243321). Following quality control, a total of 70.10 Gb of clean data was yielded. The average mapping rate of clean reads was 92.00%, indicating a high sequencing quality (Supplementary Table S3). PCA revealed that samples within the same group exhibited high similarity (Supplementary Figure S1). The intra-group correlation coefficient was robust (*p* = 0.95), whereas inter-group correlations showed significant attenuation (*p* = 0.66) (Figure 5A). These results validate the validity and reliability of the experimental data.

### 3.5. The Pathway Analysis of DEGs of Embryos at the Blastula and Gastrula Stages in Diploid and Cold Shock-Induced Groups

A substantial number of DEGs were identified in both comparison groups. At the blastula stage, 3269 DEGs were identified, with 1283 genes upregulated and 1986 genes downregulated between diploid and induced embryos. At the gastrula stage, 7183 DEGs were identified, with 3964 genes upregulated and 3219 genes downregulated (Figure 5B). The biological functions of these DEGs were further explored through GO classification and KEGG pathway analysis.

**Figure 6:**
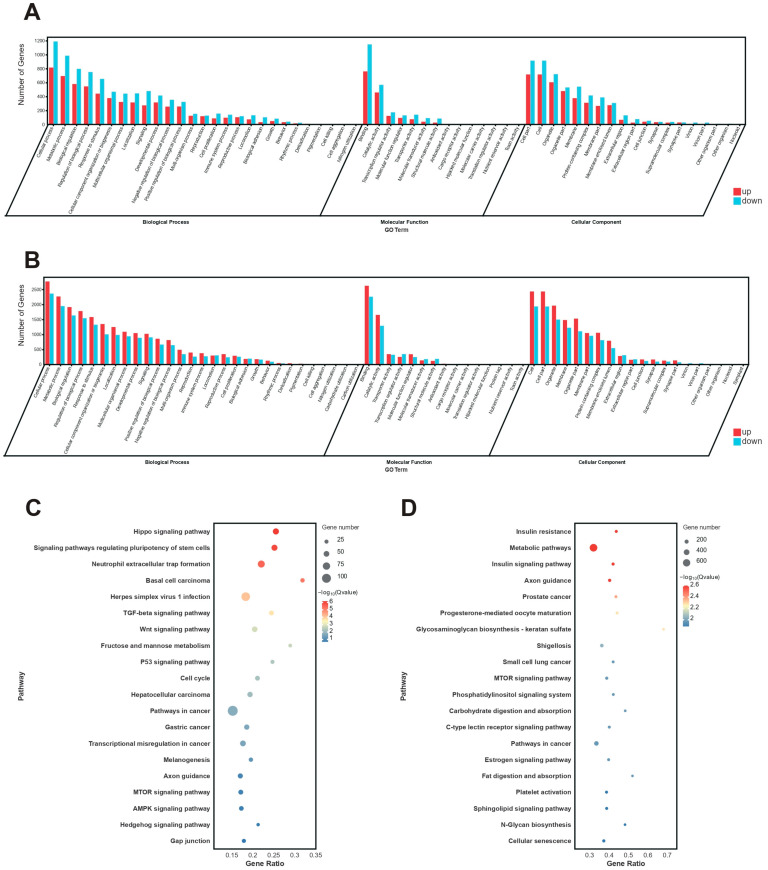
KEGG pathway enrichment and GO classifications of DEGs at the blastula and gastrula stages of diploid and cold shock-induced embryos. (**A**): GO classifications at the blastula stage. (**B**): GO classifications at the gastrula stage. (**C**): KEGG enrichment at the blastula stage. (**D**): KEGG enrichment at the gastrula stage.

At the blastula stage, DEGs were assigned to 60 GO terms, including 27 terms for biological processes (BPs), 24 terms for molecular functions (MFs), and 19 terms for cellular components (CCs). The significantly enriched processes we observed included metabolic process (GO:0008152), transcription regulation (GO:0140110), and cellular components (GO:0005623) (Figure 6A). KEGG analysis revealed significantly enriched pathways, which were associated with embryonic development, cell growth, and proliferation, including the Hippo signaling pathway (ko04390), TGF-β signaling pathway (ko04350), Wnt signaling pathway (ko04310), and p53 signaling pathway (ko04115) (Figure 6C).

At the gastrula stage, DEGs were assigned to 64 GO terms, with 29 terms for BPs, 15 for MFs, and 20 for CCs. Genes were significantly enriched in cellular processes (GO:0009987), metabolic processes (GO:0008152), binding activities (GO:0005488), and cellular components (GO:0005623) (Figure 6B). KEGG analysis revealed significant enrichment in metabolism-related pathways, including metabolic pathways (ko01100), insulin signaling pathways (ko04910), the phosphatidylinositol signaling system (ko04070), the FoxO signaling pathway (ko04068), and the mTOR signaling pathway (ko04150) (Figure 6D).

**Figure 7:**
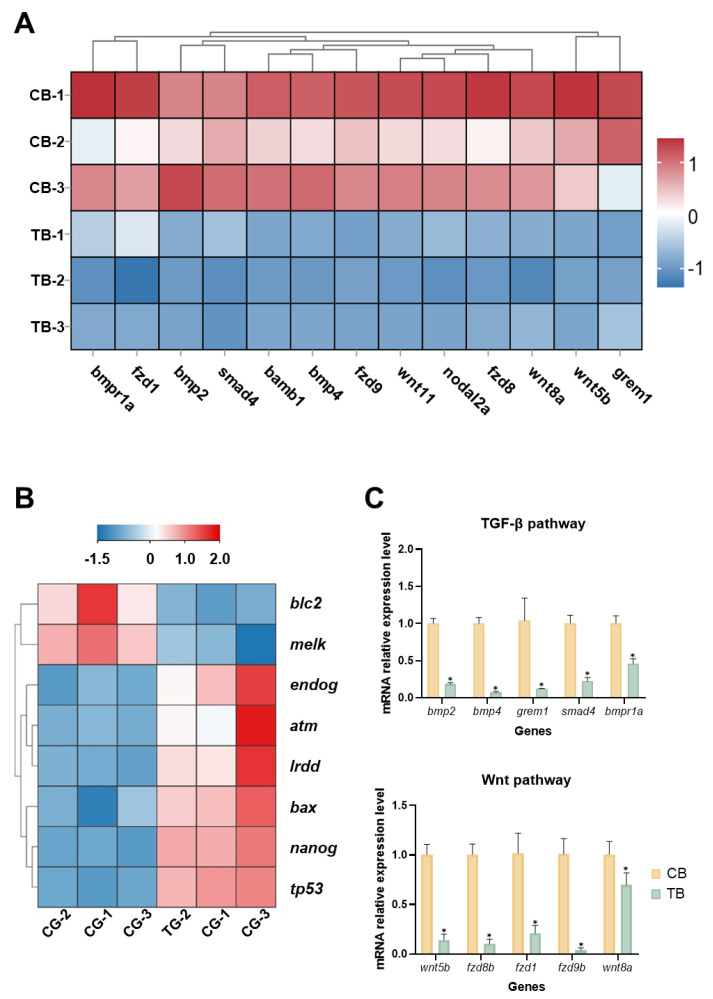
Heat map of genes and qRT-PCR validation of diploid (CB) and cold shock-induced embryos (TB). (**A**): Expression patterns of genes in the TGF-β and Wnt signaling pathways at the blastula stage. (**B**): Expression patterns of genes involved in the apoptosis signaling pathway and ZGA-related genes. (**C**): qRT-PCR validation of DEGs at the blastula stage. * present *p* < 0.05.

Notably, key components of the TGF-β signaling pathway (ko04350), such as *bmp2b*, *bmp4*, *grem1b*, *nodal2a*, *bambi*, *smad4*, and *bmpr1a*, were significantly downregulated in cold shock-induced embryos. Similarly, the expression of genes in the Wnt signaling pathway (ko04310), which play important roles in dorsal–ventral axis formation, such as *wnt5b*, *wnt8a*, *fzd8*, *wnt11*, *fzd9*, and *fzd1*, was also significantly downregulated in the induction group (Figure 7A). In addition, genes associated with zygotic genome activation (ZGA), such as *nanog* and *melk*, were significantly downregulated in cold shock-induced embryos. Moreover, genes involved in the apoptosis pathway (ko04210) exhibited significant differential expression in cold shock-induced embryos. Several pro-apoptotic genes, including *bax*, *tp53*, *atm*, and *endog*, were significantly upregulated, whereas the anti-apoptotic gene *bcl2a* was notably downregulated (Supplementary Table S5, Figure 7B).

### 3.6. Validation of DEGs with qPT-PCR

As shown in Figure 7C, qRT-PCR analysis verified that the selected DEGs exhibited expression trends comparable to those detected in the transcriptome data, supporting the robustness and reliability of the transcriptomic analysis.

## 4. Discussion

The sterility of triploid fish makes them ideal candidates for enhancing production efficiency and ensuring ecological safety [35,36]. In this study, a cold shock method was developed to induce triploid grass carp, successfully producing a substantial number of triploid fries. This method is not only convenient for operation in grass carp farms but also contributes to the genetic improvement of this species.

The goal of triploid induction is to prevent the extrusion of the PBII using physical shocks or chemical treatments. Two parameters are critical to the success of this induction process: The first is the precise timing of PBII extrusion, as the PBII is rapidly extruded after fertilization [16]. However, the timing of PBII extrusion varies across species; for example, in silver carp (*Hypophthalmichthys molitrix*), PBII extrusion occurs approximately 3 min after fertilization [37], while in zebrafish (*Danio rerio*), it happens around 10 min after fertilization [38]. A study on grass carp demonstrated that gynogenesis could be successfully induced 2 min after fertilization, indicating that PBII extrusion occurs at approximately 2 min post-fertilization [39]. Based on this observation, we initiated the cold shock 2 min after fertilization in our study. The second critical parameter is the duration of the physical shock. An insufficient shock duration fails to effectively inhibit PBII extrusion, while excessively prolonged stimulation may cause damage to the embryos [40]. In our study, 12 min cold shock treatment resulted in a triploid induction rate exceeding 70% and a hatching rate above 10%. In contrast, 10 and 14 min shock durations did not result in better triploid rates, suggesting that 12 min is the optimal treatment duration. Further research is needed to refine the induction conditions and enhance the triploid rate. A previous study investigated the induction of triploidy in grass carp via thermal shock [20]. The findings showed that cold shock treatment at 7 °C for 30 min, applied 2 min post-fertilization, resulted in an 83% triploidy induction rate, while treatment at 5 °C for 27 min, administered 4 min post-fertilization, achieved a 100% induction rate. Despite these high induction efficiencies, the proportion of viable embryos at the early-to-mid-blastula stage remained low (<10%) [20]. In addition, the triploid rate observed in our study exceeds the 8% triploid rate reported in the same study using heat shock [20], and both the fertilization and hatching rates achieved with the optimized cold shock protocol exceeded those reported in previous studies and aligned with expectations for successful triploid induction in grass carp. Although hydrostatic pressure shock has been reported to yield higher triploid rates [21], cold shock is more practical for fish farms because it does not require specialized pressure chambers, which could be a financial burden for fish farmers.

Ploidy in fish is typically determined through chromosome counting, DNA content analysis, and erythrocyte nuclear morphometry [16]. Flow cytometry is commonly employed for DNA content detection due to its convenience and sensitivity [41]. Flow cytometric analysis confirmed that the DNA content ratio of triploids to diploids was 1.5, validating the DNA content of induced triploid grass carp. Chromosome counting, the most direct and accurate method, revealed that induced triploid individuals possessed 72 chromosomes, while diploid individuals had 48 chromosomes, thus confirming the ploidy of induced triploid grass carp.

Artificially induced triploid fish generally exhibit higher mortality than diploid counterparts [42]. High mortality rates were observed in cold shock-induced embryos at the blastula and gastrula stages in our study. A similar phenomenon was reported in polyploidy studies on hybrid grouper and mandarin fish [43,44]. Various factors contribute to embryonic mortality, including anomalies in DNA repair and osmoregulation. Studies suggest that DNA damage repair and osmoregulation failures may explain the high mortality observed in hypotonic-induced triploid oyster embryos [45]. Similarly, cold shock-induced triploid hybrid groupers exhibited mortality linked to inhibited or abnormal cell division and proliferation [43]. In addition, zygotic genome activation (ZGA) is a critical developmental transition in which control shifts from maternal mRNAs to the embryonic genome, typically occurring at the mid-blastula stage in teleost fish [46]. In this study, the expression levels of key ZGA regulators, such as *nanog* and *melk*, were significantly reduced in cold shock-induced embryos, suggesting abnormal activation of the embryonic transcriptional program. Furthermore, the disruption of key regulators involved in ZGA has been shown to cause developmental delays and embryonic lethality, typically occurring at or shortly after the onset of gastrulation [47]. Therefore, abnormal ZGA may be an additional reason for the elevated mortality observed in triploid embryos.

Transcriptomic analysis revealed obvious differences in gene expression between diploid and cold shock-induced embryos at the blastula and gastrula stages. GO and KEGG analyses showed that DEGs were primarily enriched in metabolic and environmental information processing pathways. The TGF-β and Wnt signaling pathways, playing critical roles in embryonic development processes such as stem cell proliferation, differentiation, and organogenesis [48], exhibited reduced expression in the triploid induction embryos. The genes *bmp2b* and *bmp4* are critical for early embryonic development, particularly in mesoderm formation and cardiac development, with their absence resulting in embryonic lethality [49]. Similarly, *wnt8* and *wnt5b* play key roles in dorsal–ventral axis formation [50], and studies have shown that *wnt8a* deficiency leads to aberrant dorsal–ventral axis formation in zebrafish [51]. In this study, the expression of *bmp2b*, *bmp4*, *wnt5b*, and *wnt8a* was notably reduced in cold shock-induced embryos at the blastula stage. Furthermore, activation of the apoptosis pathway (ko04210) was observed in cold shock-induced embryos. Transcriptomic data revealed significant upregulation of multiple pro-apoptotic genes. The upregulation of *tp53* and *atm* is involved in DNA damage-triggered apoptotic responses, as ATM is known to activate TP53 via phosphorylation in response to genotoxic stress, leading to the transcriptional activation of downstream effectors such as BAX [52,53]. The anti-apoptotic gene *bcl2* was notably downregulated. This is consistent with previous findings demonstrating that suppression of Bcl-2 expression leads to caspase-independent autophagy and increases programmed cell death [54]. This expression profile indicates increased apoptotic signaling, potentially contributing to abnormal embryonic viability. Taken together, the concurrent downregulation of key morphogenetic genes and activation of cell death pathways provide a mechanistic explanation for the high embryonic mortality observed in triploid-induced embryos.

## 5. Conclusions

In summary, we developed a simple method for inducing triploid grass carp via cold shock, confirming the triploid characteristics through flow cytometry and karyotype analysis. Furthermore, the dynamic expression profile of induced embryos during development suggests that the high mortality observed may be attributed to the downregulation of genes in the TGF-β and Wnt signaling pathways, as well as the abnormal expression of ZGA regulators. In addition, the significant upregulation of pro-apoptotic genes and downregulation of anti-apoptotic genes indicate enhanced activation of the apoptosis pathway, which may further compromise embryonic survival. This study presents an efficient method for producing triploid grass carp and provides a molecular foundation for future investigations into the regulatory mechanisms underlying the development of artificially induced triploid embryos.

## Figures and Tables

**Table 1:** Conditions of triploid grass carp induced by cold shock treatment and the rates of fertilization, hatching, and triploid induction.

Group	Cold Temperature (°C)	Cold Shock (mpf)	Shock Duration (min)	Fertilization Rate (%)	Hatching Rate (%)	Triploid Rate (%)
1	4	2	10	61.52 ± 1.03	12.83 ± 0.77	10 ± 7.13
2	4	2	12	58.30 ± 7.54	10.06 ± 0.87	71.73 ± 5.00
3	4	2	14	52.57 ± 3.18	8.62 ± 2.87	0
Control	-	-	-	91.80 ± 1.46	80.21 ± 0.65	-

## Data Availability

Data are contained within the article.

## Supplementary Materials

The following supporting information can be downloaded at: https://www.mdpi.com/article/10.3390/ani15152165/s1, Figure S1: Principal component analysis of samples; Table S1: Primers used for qRT-PCR validation; Table S2: Embryonic development stage of cold shock-induced grass carp; Table S3: Summary of transcriptomic data obtained from the diploid and cold shock-induced embryos; Table S4: Preliminary results of triploid induction under alternative cold shock parameters (not repeatedly validated). Table S5: KEGG enrichment results for the apoptosis pathway (ko04210) during the blastula and gastrula stages.

## Abbreviations

The following abbreviations are used in this manuscript:

PBII	Second polar body
LRH-A	Luteinizing hormone-releasing hormone analogue
DOM	Domperidone
DEGs	Differentially expressed genes
GO	Gene Ontology
KEGG	Kyoto Encyclopedia of Genes and Genomes
qRT-PCR	Quantitative real-time polymerase chain reaction
BP	Biological process
MF	Molecular function
CC	Cellular component
