# Supplementary material for: Induction of Triploid Grass Carp (Ctenopharyngodon idella) and Changes in Embryonic Transcriptome
Source: Animals (Basel). 2025 Jul 22;15(15):2165. doi: 10.3390/ani15152165 (PMC12345452; doi:10.3390/ani15152165)
Supplement: Supplementary file 1 [file animals-15-02165-s001.zip › Supplementary Tables.pdf]

Name	Primer (5’ to 3’)	Tm (°C)	Amplification Efficiency (%)
bmp2	F: TCAGCGATGTTGGCTGGAAT<br>R: ATGGCGTGATTGGTGGAGTT	60	100.8
bmp4	F: CGTAAGCCGTTCCTTGCATC<br>R: ATCCAGTCGTTCCAACCCAC	60	107.6
grem1	F: AAGAGCAACCCGAACGAGTC<br>R: AGAAGGAGTTGCACTGTCCG	60	109.9
wnt5b	F: CCCTCATCGTCTGCAACTCA<br>R: CACTCCTTGATGCCCGTCTT	60	98.8
fzd8b	F: TATGAAACCACAGGTCCCGC<br>R: CCCCGCAGGTTATCCAAGTT	60	95.5
smad4	F: CTCTCCACCACCCACACTTC<br>R: AGGGGTCCACATATCCGTCA	60	108.9
fzd9b	F: GGGGCTGTGTAGCAGAAAGT<br>R: TATGACGGCAGGTGCTTTGT	60	100.9
fzd1	F: AGTCTCGCGTGTGAATCGTT<br>R: CGTGGAGCTTTTTGGGTTCG	60	102.6
wnt8a	F: AAGCCAGACATCAGCGTTCA<br>R: GTCCTCCTGGTGAAGTGTGG	60	90.8
bmpr1a	F: GATGAACACGGCGAGGGTAT<br>R: GCAAGTGGGCGTTCCATAAC	60	93.5
β-actin	F: TCCACAAGAAGGGAGCATCG<br>R: TGCACACTGCTGGTGTATGT	60	107.2

Supplementary Table S1: Primers for qRT-PCR validation.

Embryonic development	Time (hpf)
Fertilization	0
2 cell stage	0.7
4 cell stage	0.9
8 cell stage	1.1
16 cell stage	1.4
32 cell stage	1.7
64 cell stage	2.0
Early blastula stage	2.4
Mid-blastula stage	3.1
Late blastula stage	4.0
Early gastrula stage	5.4
Mid-gastrula stage	6.9
Late gastrula stage	7.6
Neurulation stage	8.3
Blastopore closure stage	9.1
Somite segmentation stage	10.4
Tail bud stage	14.7
Muscular contraction stage	16.3
Pre-hatching stage	17.9
Embryo hatching stage	21.3

Supplementary Table S2: Embryonic development stage of cold shock-induced grass carp.

Sample	Base Num (nt)	GC (%)	Q20 (%)	Q30 (%)	Num of reads mapped to genome	Mapping rate (%)
DB-1	6,051,968,898	45.60	97.76	94.02	41,524,890	90.97
DB-2	5,733,023,479	46.41	97.81	94.02	37,379,894	90.67
DB-3	5,962,211,630	47.10	97.90	94.26	37,759,020	90.65
DG-1	5,892,201,491	47.67	98.01	94.51	35,172,435	93.10
DG-2	6,129,111,679	45.98	97.76	93.95	42,958,275	92.93
DG-3	6,130,153,057	46.70	97.88	94.24	39,366,172	92.64
TB-1	5,788,066,723	47.46	98.05	94.60	39,212,411	92.29
TB-2	5,631,137,378	46.20	97.86	94.20	36,916,232	91.27
TB-3	5,825,072,907	47.15	98.04	94.56	38,865,516	93.20
TG-1	5,620,075,730	46.33	97.97	94.42	37,366,474	91.60
TG-2	5,626,730,049	46.45	97.86	94.24	37,131,282	91.93
TG-3	5,713,035,747	47.36	98.03	94.61	37,851,933	92.75
Total	70,102,788,768	-	-	-	461,504,534	-
Average	5,841,899,064	46.70	97.91	94.30	38,458,711	92.00

Supplementary Table S3: Summary of transcriptomic data obtained from the diploid and cold shock-induced embryos.

Group	Cold temperature (°C)	Cold shock (mpf)	Shock duration (min)	Triploid rates (%)
1	6	2	12	20
2	8	2	10	8.3

Supplementary Table S4: Preliminary results of triploid induction under alternative cold shock parameters (not repeatedly validated).

Group	Pathway id	Pathway	Pvalue	Qvalue
Blastula	ko04210	Apoptosis	0.020318	0.237515
Gasstrul	ko04210	Apoptosis	0.00235	0.022074

Supplementary Table S5: KEGG enrichment results for the apoptosis pathway (ko04210) during the blastula and gastrula stages.
