# Supplementary figures and images for: Induction of Triploid Grass Carp (Ctenopharyngodon idella) and Changes in Embryonic Transcriptome
Source: Animals (Basel). 2025 Jul 22;15(15):2165. doi: 10.3390/ani15152165 (PMC12345452; doi:10.3390/ani15152165)

PC1(72.1%),PC2(17.3%)

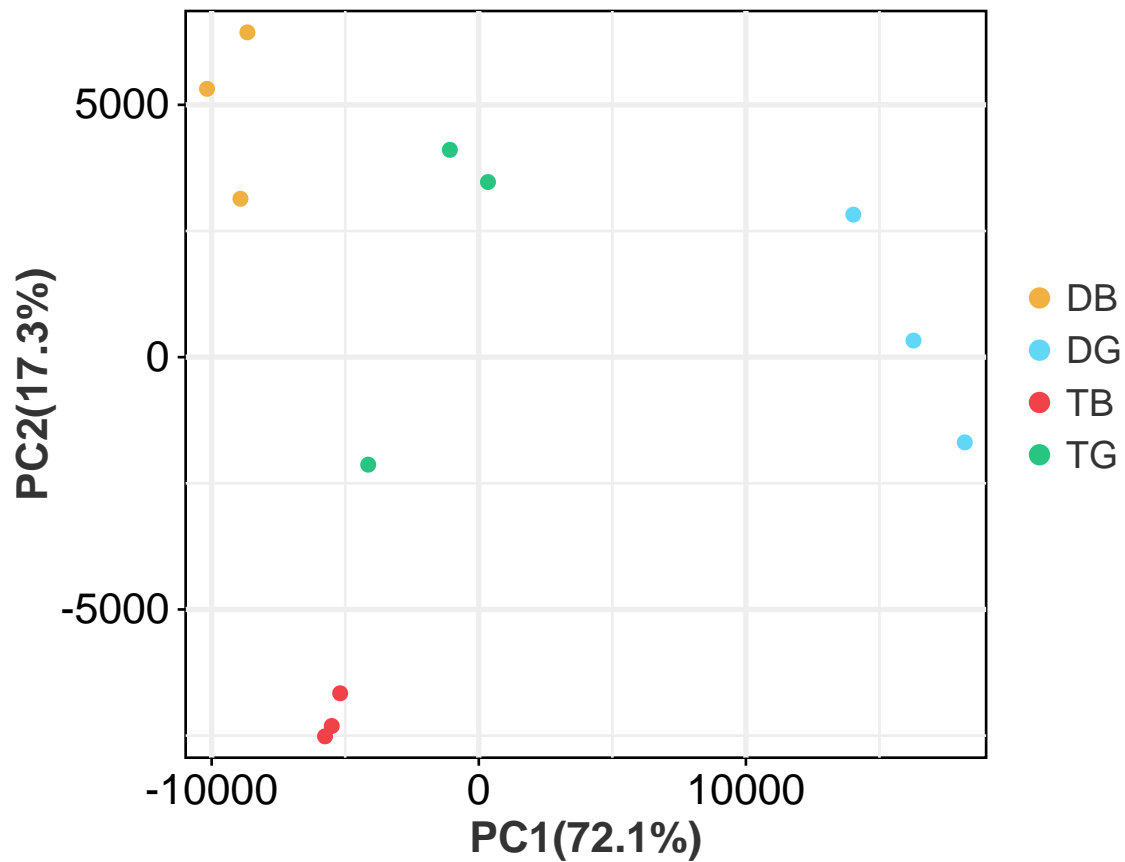

Supplement: Supplementary file 1 [file animals-15-02165-s001.zip › Supplementary Figure S1.pdf]
